# Polymer Nanomedicines with Ph-Sensitive Release of Dexamethasone for the Localized Treatment of Inflammation

**DOI:** 10.3390/pharmaceutics12080700

**Published:** 2020-07-25

**Authors:** Alena Libánská, Eva Randárová, Franck Lager, Gilles Renault, Daniel Scherman, Tomáš Etrych

**Affiliations:** 1 Institute of Macromolecular Chemistry, Czech Academy of Sciences, 162 06 Prague, Czech Republic; libanska@imc.cas.cz (A.L.); koziolova@imc.cas.cz (E.R.); 2 Institut Cochin, Université de Paris, INSERM, CNRS, 75014 Paris, France; franck.lager@inserm.fr (F.L.); gilles.renault@inserm.fr (G.R.); 3 Unité de Technologies Chimiques et Biologiques pour la Santé, Faculté de Pharmacie, Université de Paris, CNRS UMR 8258, Inserm U1267, 75270 Paris, France; daniel.scherman@parisdescartes.fr

**Keywords:** polymer conjugate, drug delivery, inflammation, HPMA, dexamethasone, adjuvant-induced arthritis, passive targeting

## Abstract

Polymer-drug conjugates have several advantages in controlled drug delivery to inflammation as they can accumulate and release the drug in inflamed tissues or cells, which could circumvent the shortcomings of current therapy. To improve the therapeutic potential of polymer-drug conjugates in joint inflammation, we synthesized polymer conjugates based on *N*-(2-hydroxypropyl) methacrylamide) copolymers labeled with a near-infrared fluorescent dye and covalently linked to the anti-inflammatory drug dexamethasone (DEX). The drug was bound to the polymer via a spacer enabling pH-sensitive drug release in conditions mimicking the environment inside inflammation-related cells. An in vivo murine model of adjuvant-induced arthritis was used to confirm the accumulation of polymer conjugates in arthritic joints, which occurred rapidly after conjugate application and remained until the end of the experiment. Several tested dosage schemes of polymer DEX-OPB conjugate showed superior anti-inflammatory efficacy. The highest therapeutic effect was obtained by repeated i.p. application of polymer conjugate (3 × 1 mg/kg of DEX eq.), which led to a reduction in the severity of inflammation in the ankle by more than 90%, compared to 40% in mice treated with free DEX.

## 1. Introduction

Chronic inflammatory diseases (CID), such as rheumatoid arthritis (RA), osteoarthritis (OA) or asthma, have a substantial socio-economic impact, high prevalence, and severely limit patients’ quality of life [1]. Although clinical symptoms of diverse CID vary, cellular processes in chronically inflamed tissues are similar [2]. In particular, RA can lead to severe disability and premature mortality [3,4]. In RA, the inflammation is polyarticular, primarily affecting the small diarthrodial joints of the hands and feet. However, any joint can be affected and severe non-joint systemic symptoms may occur [5]. Nonsteroidal anti-inflammatory drugs (NSAIDs), corticosteroids, disease-modifying anti-rheumatic drugs (DMARDs), and biologicals are used for the long-term treatment of RA [6] but their application does not usually lead to complete remission. In addition, the sustained administration of current anti-rheumatic agents might induce severe side effects, e.g., gastric ulceration, osteoporosis, or hematotoxicity. Moreover, these drugs exhibit additional disadvantages, e.g., low solubility, bioavailability and poor joint penetration, degradation by GI enzymes, first-pass metabolism, or food interactions [7].

In recent years, several nanomedicine approaches for enhanced RA treatment have been proposed [8,9,10]. Among them, the *N*-2-(hydroxypropyl)methacrylamide copolymers (pHPMA) may serve as efficient and safe drug carriers capable of passive targeting to inflamed joints. Their accumulation in the inflammatory site is attributed to the Extravasation through Leaky Vasculature and subsequent Inflammatory cell-mediated Sequestration (ELVIS) in the arthritic joints [11]. The main benefits of the HPMA polymers lie in their biocompatibility, non-toxicity, non-immunogenicity, and ability to prolong the circulation of the drug in the organism [12]. The HPMA-based polymer conjugates with anti-inflammatory drug dexamethasone (DEX) have proved to be more efficient in collagen-induced arthritis in rats than free DEX or DEX-loaded liposomes [13,14], with DEX attached to water-soluble HPMA copolymers via a biodegradable spacer enabling the drug release in the affected joints [15]. However, in these works the DEX release from the polymer was very slow (up to 7% per week), thus, the anti-inflammatory activity was achieved only at very high doses (60 mg/kg of DEX equivalent in arthritic mice [16]), since the blood half time of the HPMA-based polymer conjugates with a molar weight under renal limits is up to 35 h [17,18,19]. Nevertheless, for acute states of inflammation or severe exacerbation of CID where high initial doses of anti-inflammatory drugs are necessary, the application of the polymer conjugate with very slow drug release may not be suitable.

Therefore, in the present work, we developed biocompatible pHPMA-DEX conjugate with a fast pH-sensitive release intended for targeted and intensive treatment of various severe states of inflammation. As a proof of concept, we studied the in vivo biodistribution and therapeutic activity of pHPMA-DEX conjugates labeled with a near-infrared fluorescent dye using adjuvant-induced arthritis in mice. The DEX was linked to the pHPMA via tailored spacer enabling direct attachment and subsequent fast pH-sensitive drug release at the inflammation site to achieve rapid inflammation suppression at a reduced dose of DEX conjugates with minimal side effects. The synthesis of polymer precursors was based on the controlled RAFT polymerization of HPMA and methacrylamide-based comonomers and consecutive attachment of bioactive molecules to prepare highly defined polymer-based drug delivery systems.

## 2. Materials and Methods

### 2.1. Materials

Tert-butanol (*t*-BuOH), dimethyl sulfoxide (DMSO), diethyl ether, ethyl acetate, acetone, methanol (MeOH), dimethylacetamide (DMA), 2,2′-azobis(isobutyronitrile) (AIBN), 2,2′-azobis(4-methoxy-2,4-dimethylvaleronitrile) (V70), acetic acid (CH_3_COOH), dexamethasone (DEX), 4-(2-oxopropyl)benzoic acid (OPB), N-(3 dimethylaminopropyl)-N′-ethylcarboiimide hydrochloride (EDC), 4-(dimethylamino)pyridine (DMAP), dimethylformamide (DMF), dichloromethane (DCM), and porcine liver esterase were obtained from Merck KGaA (Darmstadt, Germany). The fluorescent dyes cyanine 5.5-succinimidyl ester (Cy5.5-NHS ester) and cyanine 5.5 (Cy 5.5) were obtained from Lumiprobe GmbH, (Hannover, Germany). Milli-Q water (H_2_O) was used for all experiments and obtained from the appliance Millipore Merck, Darmstadt, Germany.(resistivity 18.2 MΩ·cm, 25 °C, organic carbon ≤ 5 ppb). Dexamethasone (2 mg/mL of excipient QSP) used in in vivo experiments was purchased from Vibrac S.A. (Carros, France) and 3-aminophthalhydrazide monosodium salt was obtained from Thermo Fisher GmbH (München, Germany). Complete Freund’s adjuvant (CFA) was obtained from Becton, Dickinson and Company, Franklin Lakes, NJ, USA.

### 2.2. Synthesis of Monomers

N-(2-hydroxypropyl)methacrylamide (HPMA) and N-(terc-butoxycarbonyl)-N′-(6-methacrylamidohexanoyl)hydrazine (Ma-Ah-NHNH-Boc) were prepared according to the literature [20]. Prepared monomers were characterized by elementary analysis (calc./found) and high-performance liquid chromatography (HPLC): HPMA (purity ˃ 99.8%; C 58.72/58.98, H 9.15/9.18, N 9:15/9.82) a Ma-Ah-NHNH-Boc (purity ˃ 99.5%; C 57.70/57.96, H 8.33/8.64, N 13.46/13.25).

### 2.3. Synthesis of Trithiocarbonate Chain Transfer Agent (CTA)

The trithiocarbonate chain transfer agent S-2-cyano-2-propyl-S′-ethyl trithiocarbonate (AIBN-TTc) was synthesized as described by Ishitake et al. [21]. The HPLC showed a single peak with a retention time of 10.7 min, 1H NMR, d: 1.36 (t, 3H, SCH2CH3), d: 1.88 (s, 6H, C(CH3)2CN), d: 3.35 (q, 2H, SCH2CH3).

### 2.4. Synthesis of HPMA Linear Copolymer Precursor

The linear copolymer precursor (LC-TTc) was prepared by controlled radical Reversible Addition Fragmentation Chain Transfer (RAFT) copolymerization of HPMA and Ma-Ah-NHNH-Boc in a molar ratio 92:8 using AIBN-TTc as CTA and AIBN as an initiator. The copolymerization was carried out in a mixture of 85% of t-BuOH and 15% of DMA. The molar ratio of monomers:V70:TTc-AIBN was 500:2:1. The LC-P was prepared as follows: the monomer HPMA (1.00 g, 6.98 mmol) was dissolved in t-BuOH (8.1 mL) and the Ma-Ah-NHNH-Boc (0.19 g, 0.607 mmol) in DMA (0.6 mL), then the solution of AIBN-TTc (5.20 mg, 20.53 µmol) and V70 (9.37 mg, 30.04 µmol) in DMA (0.8 mL) was added into the solution of monomers. The copolymerization mixture was inserted into an ampule, bubbled with argon for 10 min, and sealed. The copolymerization was conducted at 30 °C for 72 h, with the copolymer isolated by precipitation into a mixture of acetone:diethyl ether (2:1), filtered, and dried in a vacuum, yielding 0.92 g (77%).

The trithiocarbonate ω-end group was removed using 2,2′-azobisisobutyronitril (AIBN) as described in the literature [22]. LC-TTc (780 mg, 3.2 mmol of TTc) and AIBN (156 mg, 0.97 mmol) were dissolved in 7 mL of DMA (10% solution). The reaction mixture was inserted into an ampule, bubbled with argon for 10 min and sealed. The reaction was carried out for 3 h at 80 °C, with the final copolymer (LC) isolated by precipitation into a mixture of acetone:diethyl ether (2:1), filtered and dried in a vacuum.

The deprotection of the hydrazide groups was performed in distilled water at 100 °C as described in the literature after the removal of the TTc end groups [23]. The copolymer LC (650 mg) was dissolved in 5850 μL of distilled water, placed in an ampule, bubbled with argon for 10 min, sealed, and heated for 1 h to 100 °C. The final copolymer (LC-NHNH_2_) was separated by freeze-drying.

### 2.5. Synthesis of the Copolymer Conjugate with The Fluorescent Dye (LC-Cy5.5)

The fluorescently labeled polymer conjugate was prepared by conjugation of LC-NHNH_2_ with free hydrazide with the fluorescent dye Cy 5.5-NHS ester-forming a stable hydrazide bond. LC-NHNH_2_ (220 mg) and Cy 5.5 NHS ester (4.4 mg, 5.7 µmol) were dissolved in 2 mL of MeOH (10% solution) and the mixture was left to react at 37 °C for 12 h. The unreacted free fluorescent dye was separated on a Sephadex LH-20 column in MeOH, with the polymer conjugate isolated by precipitation into ethyl acetate, filtered, and dried in a vacuum. The reaction course was observed using an HPLC analyzer equipped with an SPD-M20A photodiode array detector (Shimadzu, Japan) using a reverse-phase column Chromolith Performance RP-18e (100 × 4.6, eluent water acetonitrile with acetonitrile gradient 0–100 vol%, flow rate 5 mL/min).

### 2.6. Synthesis of Copolymer Conjugate with the Derivate of Dexamethasone (LC-DEX-OPB or LC-Cy5.5-DEX-OPB)

The 4-(2-Oxopropyl) benzoic acid ester of DEX (DEX-OPB) was synthesized and characterized as described previously in the literature [24].

The polymer conjugate (LC-DEX-OPB) and the fluorescently labeled polymer conjugate (LC-Cy5.5-DEX-OPB) with the derivate of dexamethasone were synthesized by conjugation of the appropriate copolymers with DEX-OPB forming a pH-sensitive hydrazone bond. The deprotected LC-NHNH_2_ or LC Cy5.5 (50 mg) and DEX-OPB (1.8 mg, 2.5 µmol) were dissolved in 1.25 mL of MeOH and 50 μL of CH_3_COOH was added to the solution. The mixture was left to react for 2 h at room temperature. The unreacted free DEX-OPB was separated on a Sephadex LH-20 column in MeOH. The polymer conjugate was isolated by precipitation into ethyl acetate, filtered, and dried in a vacuum.

### 2.7. Characterization of the Polymer Carriers and the Conjugates

The number-average molecular weights (*M*_n_), weight-average molecular weights (*M*_w_), and the dispersity (*Đ*) of the polymer precursor and conjugates were measured using size-exclusion chromatography (SEC) on an HPLC Shimadzu system equipped with an SPD-M20A photodiode array detector (Shimadzu, Japan), an OptilabrEX differential refractometer and a multiangle light scattering DAWN HELEOS II (Wyatt Technology, Santa Barbara, CA, USA) detector using 0.15 M sodium acetate buffer at pH 6.5 (20%) and methanol (80%, *v/v*) as the mobile phase. TSKgel SuperSW3000 or the combination of the AW3000 + AW4000 column was used. The ASTRA software Version 5.3 and the refractive index increment dn/dc = 0.167 mL/g were used for calculations.

The content of hydrazide groups was determined by UV/VIS spectrophotometry after derivatization using TNBSA as previously described [23]. The content of TTc end groups was determined spectrophotometrically at 305 nm and was used for calculations of the polymer functionality. Molar absorption coefficient was ε_305,TTc_ = 11,200 L·mol^−1^·cm^−1^.

The total content of DEX was measured by HPLC. The sample was dissolved in the mobile phase of water:acetonitrile 95:5 with 0.1% of trifluoracetic acid and incubated for 1 h at 37 °C. The Chromolith reverse-phase column was used for HPLC. The content of the Cy5.5 was measured by UV/Vis spectrophotometry in methanol using the molar absorption coefficient ε_670_ = 209,000 L.mol^−1^.cm^−1^ estimated for Cy5.5-NHS ester.

The hydrodynamic radius (*R*_H_) of the polymer precursors was measured with a Nano-ZS instrument (ZEN3600, Malvern, UK) in a phosphate buffer (pH 7.4, 0.1 M with 0.05 M NaCl). The concentration of the polymer was 5 mg/mL. The intensity of the scattered light was detected at an angle θ = 173° using a laser with a wavelength λ = 632.8 nm. The DTS (Nano) program was used for the dynamic light scattering data evaluation. The values are equivalent to the mean of at least five independent measurements. The values were not extrapolated to the zero concentration.

### 2.8. In Vitro Drug Release

The in vitro drug release kinetics of DEX-OPB from LC-DEX-OPB was evaluated at pH = 5.0, 6.3, and 7.4 (0.1 M phosphate buffer) at 37 °C. The concentration of the conjugate LC DEX-OPB in the solution was 1.5 mg/mL. At predetermined time intervals, 200 μL of the solution was withdrawn, extracted with chloroform (800 μL), then the chloroform phase (600 μL) was withdrawn and evaporated. The released DEX-OPB was redissolved in 100 μL of MeOH, then analyzed with an HPLC analyzer (Shimadzu, Japan) using a reverse-phase Chromolith Performance RP-18e column (100 × 4.6, eluent water acetonitrile with acetonitrile gradient 0–100 vol.%, flow rate 5 mL/min) with UV detection at 240 nm.

The in vitro drug release kinetics of DEX-OPB and DEX was also evaluated in the presence of porcine liver esterase. First, the activity of the enzyme was established spectroscopically by the reaction of the enzyme with the substrate 4-methylumbelliferyl acetate. This reaction generates the product 4-methylumbelliferon which can be determined spectroscopically (λ = 350 nm, ɛ_350_ = 12,200 L·mol^−1^·cm^−1^). The substrate (1.98 mg) was dissolved in 367 μL of DMSO (0.025 M) and the solution was tempered to 37 °C. The esterase was dissolved in the 0.05 M phosphate buffer (pH 7.4, 37 °C) at a concentration of 0.3 mg/mL. The solutions were inserted in the 1 cm cuvettes: 970 μL of phosphate buffer, 20 μL of the substrate, and 10 μL of the solution of the enzyme. The reference value of the absorbance was set by dosing of 980 μL of phosphate buffer and 20 μL of the substrate. Cuvettes were tempered and the measurement was performed at 37 °C. The specific activity of the enzyme was 6.8 mmol.min^−1^ mg^−1^.

For the degradation study, the concentration of the enzyme 0.3 μg/mL was according to the literature [25] and the concentration of the polymer was 1 mg/mL. LC-DEX-OPB (200 μL) dissolved in phosphate-citrate buffer, pH 5 (1 mg/mL) was incubated with 10 μL of the esterase solution (phosphate buffer, pH 7.4, 0.3 μg/mL) at 37 °C. At predetermined time intervals, the sample solution was extracted with chloroform (800 μL), then 600 μL of the chloroform phase was withdrawn and evaporated. The extracted DEX-OPB was dissolved in 100 μL of MeOH, then analyzed with an HPLC analyzer (Shimadzu, Japan), using a reverse-phase Chromolith Performance RP-18e column (100 × 4.6, eluent water acetonitrile with acetonitrile gradient 0–100 vol%, flow rate 5 mL/min) with UV detection at 240 nm.

### 2.9. In Vivo Animal Model—Adjuvant-Induced Acute Arthritis (AIA)

Five week old male DBA/1 mice were purchased from Janvier Labs (Saint Berthevin Cedex, France) and allowed to acclimatize for at least 1 week. Mice were fed with a special diet without chlorophyll for at least 5 days before the start of the experiment (Day 0) to minimize the nonspecific background fluorescent signal from the intestine. AIA, an acute articular inflammation mouse model, was induced by a single intra-articular injection of 10 to 20 µL of complete Freund’s adjuvant into the right ankle joint of each mouse. One mL of CFA contains 1 mg of Mycobacterium tuberculosis-H37Ra, heat-killed and dried, 0.85 mL paraffin oil, and 0.15 mL mannide monooleate. [26] Weight variation, clinical severity (CS) of inflammation status, and fluorescence from mice were observed daily. Mice were sacrificed after 72 h of observation due to ethical reasons. All animal experiments followed European and French animal experimentation regulations. The project was approved by the local Ethics Committee (CEEA 34, Université Paris Descartes, 5 September 2017 and registered by the French Ministry of Research (number 20123-2019021516441070).

### 2.10. Clinical Assessment of Arthritis

Mice were monitored for evidence of arthritis in their four paws using a blind procedure by two examiners (A.L. and F.L.) and a CS based on disease severity was given for each mouse. Clinical assessment was performed every day after the injection of the polymer conjugate. Briefly, the date of disease onset was recorded, and the CS of each joint or group of joints (AIA: tarsus, ankle) was graded as follows: 0 (normal, no evidence of erythema and swelling), 1 (erythema and mild swelling or local redness confined to the tarsal or ankle joint), 2 (erythema and mild swelling extending from the ankle to tarsals), 3 (erythema and moderate swelling extending from the ankle to metatarsal joints) or 4 (erythema and severe swelling encompass the ankle, foot, and digits, or ankylosis of the limb or necrosis). The mean arthritic score on each clinical observation day was calculated in each group [27,28,29].

### 2.11. In Vivo and Ex Vivo Biodistribution of Fluorescently Labeled Polymer Conjugates

A Fluobeam-700^®^ camera (Fluoptics, Grenoble, France) (690 nm excitation wavelength, emission over 700 nm, with a field set to 12.8 × 9.4 cm^2^) was used to observe the biodistribution of the fluorescently labeled polymers, LC-Cy5.5, and LC-Cy5.5-DEX-OPB, and free dye. The polymer samples were administered into the mice (6/group) one day after arthritis induction (Day 1). The polymer was dissolved in PBS at a concentration of 0.1 mg of Cy5.5/kg (200 µL/mouse) and injected i.v. via the tail vein or i.p. for LC-Cy5.5-DEX-OPB. The biodistribution of the fluorescently labeled polymer was observed at eight-time points (T_0_ = 0 h, T_1_ = 0.25 h, T_2_ = 1 h, T_3_ = 3 h, T_4_ = 6 h, T_5_ = 24 h, T_6_ = 48 h, T_7_ = 72 h) using the Fluobeam-700^®^ system. Moreover, during the biodistribution experiment, the mice were also subjected to in vivo therapeutic efficacy evaluation with clinical observation described in detail in the section above.

Free fluorescent dye Cy 5.5 (excitation maximum 684 nm, emission maximum 710 nm, molecular weight 767.66 g/mol) was used as a control. The injected Cy5.5 solution in PBS with 0.1% DMSO had a fluorescent dye concentration of 0.1 mg/kg and 200 µL was injected once i.v. to each mouse. Mice were sedated using isofluorane in air (4% for induction, 2% to maintain anesthesia, using a Minerve équipement vétérinaire system-Esternay, Esternay, France). They were then attached to the heating pad in a supine position, with the hair removed from the knee, ankle, and belly using a depilatory cream. Hind paws were positioned with the adapted orientation toward the camera using ultrasound contact gel. The working distance of the Fluobeam^®^ from the anesthesia plate was set to 15 cm.

For the ex vivo biodistribution study, the mice were sacrificed at day 4 by cervical dislocation, and the organs of interest were dissected (spleen, kidneys, liver, lungs, heart, left knee, right knee, left paw, right paw). The organs were weighed and their fluorescence was measured using the Fluobeam-700^®^. The obtained images were evaluated using the MacBiophotonic ImageJ computer program (Macbiophotonics, Hamilton, Canada). A region of interest (ROI) was drawn around each site showing the fluorescence signal: paw right, paw left, knee right, knee left, bladder, liver. The same ROIs were used in every image to ensure that the same surface was used for quantification of the fluorescent signal. From these ROIs, the software calculated the integrated density (IntDens) obtained by summing all the pixels within a region to give a total value, and the mean of the gray value of the pixels (Mean).

In vivo signals from the left and right paw were evaluated according to the formula (Mean_right paw_/Mean_left paw_) and ex vivo signal (IntDens_organ_/Weight_organ_)/(IntDens_left paw_/Weight_left paw_). The values were normalized to the control left paw (non-arthritic), the average values of these parameters are displayed. The results were normalized to the laser beam intensity.

### 2.12. In Vivo Therapeutic Effect of DEX-OPB Polymer Conjugate

For the therapeutic experiment, six DBA/1 mice were treated with LC-Cy5.5-DEX-OPB, with two groups of six DBA/1 mice treated with DEX or injected with PBS used as a control.

Two experiments with different dosing were performed. In the first experiment, 3 mg/kg of DEX equivalent (DEX eq.) was injected i.p. to avoid fast elimination from the organism observed in i.v. injections [30]. The DEX eq. injected volume was set up to 200 µL. A volume of 200 µL of linear copolymer LC-Cy5.5-DEX-OPB was administrated i.v. (via tail vein) or i.p. In the second experiment, 1 mg/kg of DEX equivalent was used. These doses were administrated i.p. once a day for three days for DEX and once a day for three days i.p. or i.v. for LC-Cy5.5-DEX-OPB.

Myeloperoxidase activity from neutrophils was monitored using in vivo bioluminescence imaging of luminol [31] in the hind paw daily (T_0_ = 0 h, T_1_ = 24 h, T_2_ = 48 h, T_3_ = 72 h) using a bioluminescence imaging system Photon Imager RT (Biospacelab, Nesles la Vallée, France). Briefly, animals were anesthetized using isoflurane (see above) and placed in the imaging system in a supine position with hind paws attached to the imaging plate using ultrasound contact gel to maintain this position during image acquisition. Mice were then injected intraperitoneally with 300 mg/kg of luminol (3-aminophthalhydrazide monosodium salt substrate dissolved in 0.9% saline solution, Alfa Aesar, Kandel, Germany). The acquisition was performed 3 min after injection and photons were collected by the imaging system for 10 min. The Photon Imager’s intensified Charge-Coupled Device (iCCD) camera performs a real-time count of photons and allows us to generate for each ROI drawn the time course of mean photon intensity emission during the 10-min acquisition. Quantification of photon intensity was performed using the dedicated image analysis software M3 Vision (Biospacelab, Nesles la Vallée, France). Each ROI was placed encompassing hind paws, keeping the same surface in cm². The software calculated the mean photon intensity expressed as photons per second per steradian, (ph/s/sr) from the average time curve generated for every ROI. A reference ROI was drawn in an untreated mouse area to measure background noise in each mouse.

### 2.13. Statistical Analysis

Values are presented as the average of at least three measurements. The standard deviation two-way analysis of variance (ANOVA) was performed, followed by a post hoc test (Bonferroni’s test) for multiple comparisons using Prism software (GraphPad, USA). A value of *p* ≤ 0.0001 was considered as statistically significant (****) and a value of *p* ˃ 0.05 as nonsignificant. The values between these two were labeled as *** for *p* ≤ 0.001, ** for *p* ≤ 0.01, and * for *p* ≤ 0.05.

## 3. Results and Discussion

### 3.1. Design and Synthesis of Anti-Inflammatory Polymer-Based Therapeutics

#### 3.1.1. Synthesis of Linear Copolymer Precursors and Carriers

The linear copolymer precursor LC-TTc was synthesized by the RAFT copolymerization of HPMA and MA-Acap-NHNH-Boc to obtain polymer precursors with very low dispersity (Figure S1). The trithiocarbonate-based CTA AIBN-TTc and low-temperature initiator V70 were used since their utilization provides high yields of well-defined HPMA copolymers. The ratio between monomers and CTA was chosen to obtain a suitable molar mass, which is below the renal filtration threshold of HPMA-based polymers. The prepared well-defined copolymers with required molar mass (*M*_w_ = 33 kg/mol) exhibited very low dispersity (*Đ* = 1.05) and high-end group functionality (*F*_TTc_ = 0.98), thus proving the controlled polymerization mechanism, which is in a good agreement with our previous results, where the linear HPMA copolymers were prepared by RAFT polymerization [23].

The reactive trithiocarbonate end functional group was removed using AIBN according to Perrier, thus reaching LC [22]. This removal is crucial to prevent the potential problems caused by the labile C–S bond of the TTc group in further reactions and applications of formed polymers. After TTc removal, the dispersity remained the same and successful TTc removal was confirmed by the disappearance of absorbance at 305 nm (characteristic for TTc group) via UV/Vis spectroscopy (Figure S2).

The final step in the preparation of the copolymer precursor was the deprotection of the hydrazide functional groups (LC-NHNH_2_) using a simple and effective procedure under mild conditions [32]. During this reaction, complete deprotection occurred as proved by NMR. Moreover, the lyophilization process led to complete purification of the polymer carrier because the side products of deprotection reaction, i.e., isobutylene and carbon dioxide, are gaseous. The characteristics are summarized in Table 1.

#### 3.1.2. Synthesis of Copolymer Conjugates with a Fluorescent Dye and Derivate of Dexamethasone

The labeled linear polymer was prepared by reaction of the deprotected hydrazide functional group with the NHS ester functional group of the fluorescent dye Cy5.5. This reaction forms a hydrazide bond stable at physiological pH, ensuring that the dye will not be released from the polymer chain in vivo. The fluorescent dye was bound in an amount of 1.75 wt% or 1.00 wt%, which is sufficient for observing the fluorescence in vivo. The accurate *M*_w_ and *Đ* could not be calculated due to the interference of drug/dye fluorescence with the laser wavelength, nevertheless, GPC profiles of the fluorescently labeled polymer were similar to those of the corresponding polymer precursor.

The DEX derivative (DEX-OPB) was synthesized by the coupling of DEX and OPB to obtain the derivative with a proper functional group for tailored pH-sensitive binding. The linear copolymer conjugates with DEX-OPB were prepared by reaction of the free hydrazide functional groups of the polymer chain LC-Cy5.5 or LC-NHNH_2_ and keto functional group of the drug derivate. The amount of dexamethasone derivate was 6.0 wt% for the sample LC-Cy5.5-DEX-OPB (further used for in vivo tests) and 5.2 wt% for the LC-DEX-OPB (further used for in vitro test). The characteristics are summarized in Table 2. These two linear copolymer conjugates, LC-Cy5.5, and LC-Cy5.5-DEX-OPB, were used in in vivo study of biodistribution and therapeutic activity. The reaction scheme of the synthesis of the conjugates is displayed in Figure S1.

#### 3.1.3. In Vitro Dexamethasone Release

**Figure 1:**
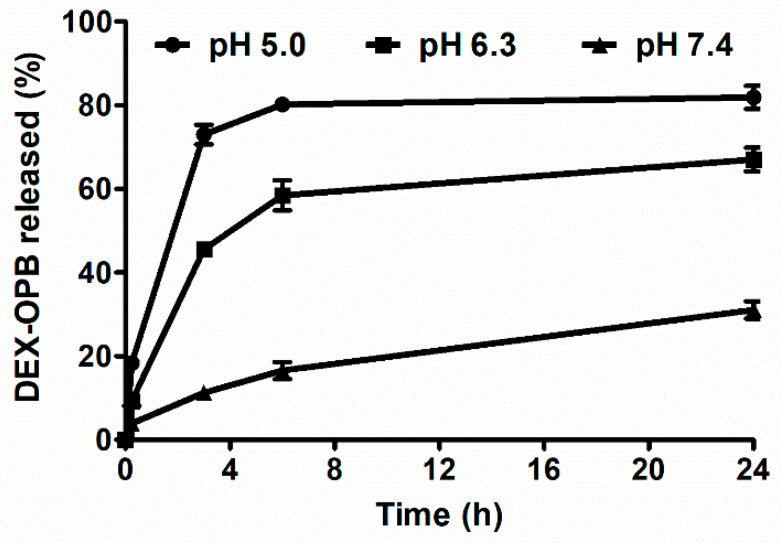
Release of dexamethasone (DEX)-4-(2-oxopropyl)benzoic acid (OPB) at pH = 5.0, pH = 6.3, pH = 7.4.

The anti-inflammatory drug DEX was bound to the polymer carrier via the pH-sensitive hydrazone bond, which enables drug release in arthritic tissue, where the pH is slightly acidic. The pH-sensitive drug release was demonstrated in vitro in buffers of pH = 5.0; 6.3; 7.4, see Figure 1. The buffers were chosen as a model environment of the blood vessel (pH = 7.4), inflammatory tissue (pH = 6.3) and intracellular lysosomes (pH = 5.0).

The release rates of DEX-OPB from our polymer precursors prepared by the RAFT technique are in good agreement with previous reports, where the polymer carrier was prepared by free radical copolymerization [33]. As expected, DEX-OPB release was much faster at acidic pH = 5.0 and pH = 6.3 than at physiological pH. Most DEX-OPB (80%) was released at pH = 5.0 within 6 h when the drug release curve reached its plateau, which is caused by the equilibrium in the closed experiment setting. Similarly, at pH = 6.3, the rapid release of DEX-OPB (60%) was observed within 6 h, thus showing the activatable properties of LC-DEX-OPB at the site of inflammation, whereas at pH 7.4, the hydrazone bond between the polymer carrier and DEX-OPB was considerably more stable. Within 6 h, less than 16% of DEX-OPB was released and until the end of the experiment (96 h) up to 52% of the DEX-OPB remained linked to the polymer. The release of the DEX itself was not observed. The ester bond between DEX and OPB was not hydrolyzed in phosphate buffers of different pH within the tested period. The fast DEX-OPB release, especially in an acidic environment, is favorable since the time of the adjuvant-induced arthritis model injected intra-articullary is very short (5 days) and slower drug release would presumably lead to minimal therapeutic effects. In addition, since the maximal joint accumulation of the prepared linear HPMA copolymers occurs within 6 h (see results of in vivo biodistribution in the Section 3.2.1), a relatively fast drug release in the inflammatory joint is required. No differences in the rate of release were observed while plasma was applied instead of the buffer of pH 7.4.

**Figure 2:**
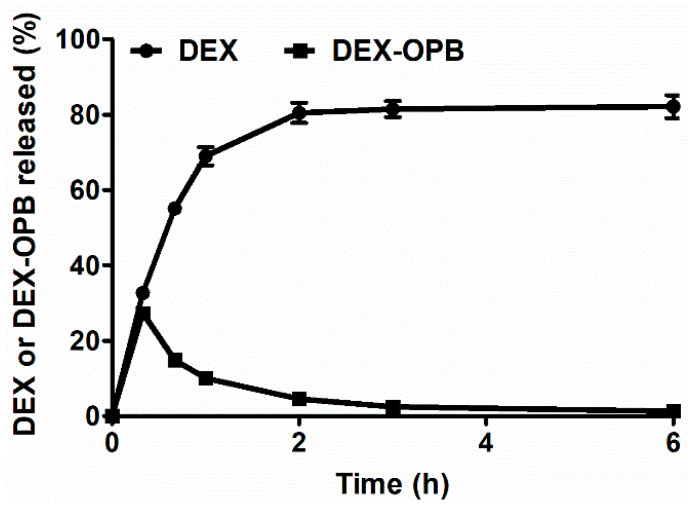
Release of the DEX-OPB/DEX in the presence of carboxylesterase.

The release rate of the drug from the polymer carrier was also studied in the presence of enzyme carboxyesterase, thus mimicking the transformation rate of DEX-OPB to DEX after uptake of polymer conjugate to inflammatory cells. The experiment was conducted at pH 5.0 since carboxyesterase is present and effective in the acidic lysosomal environment [34]. The release of both the drug derivative DEX-OPB and the drug DEX from the polymer conjugate LC-DEX-OPB was observed. The carboxyesterase catalyzes ester bonds hydrolysis [35], in the present case, the hydrolysis of the DEX-OPB to DEX and OPB. The reaction was monitored by HPLC and we observed the presence of all mentioned substances, i.e., DEX-OPB, DEX, and OPB, in the sample almost immediately after carboxyesterase addition. After 1 h, most DEX-OPB was hydrolyzed to DEX, and a negligible amount of DEX-OPB remained present in the sample (Figure 2). Therefore, drug release at pH 5 is up to three times faster in the presence of carboxyesterase. Most of the DEX-OPB was released from the polymer within 3 h at pH 5.0 and the drug was directly transformed to DEX by carboxyesterase. This suggests that fast metabolization of DEX-OPB after endosomal uptake in secondary lysosomes might occur, thus a similar therapeutic effect of DEX-OPB compared to DEX should be expected.

### 3.2. In Vivo Study

#### 3.2.1. In Vivo Biodistribution of Polymer Conjugates

The linear HPMA-based copolymer with the precursor *M*_w_ around 35 kg/mol was selected for the biodistribution study, since its *M*_w_ is under the renal threshold and it can be fully eliminated from the blood circulation by kidney glomerular filtration [36], preventing undesired long-term polymer persistence in the body. The strong fluorescence signal from the bladder, which is visible until 24 h after injection of LC-Cy5.5, proves the polymer elimination by kidneys. To quantify polymer accumulation in arthritic joints, arthritis was induced in the right hind joint by intra-articular injection of complete Freund’s adjuvant, and the left-hind joint served as a control. The polymer accumulation at the inflamed site occurred very rapidly, within 15 min after administration, and lasted until the end of the experiment. Despite most polymer in the bloodstream being excreted by kidneys within 24 h, the higher fluorescent signal in the right paw after 48 h and 72 h compared to the healthy left paw supports the premise of polymer retention in the inflamed joints.

**Figure 3:**
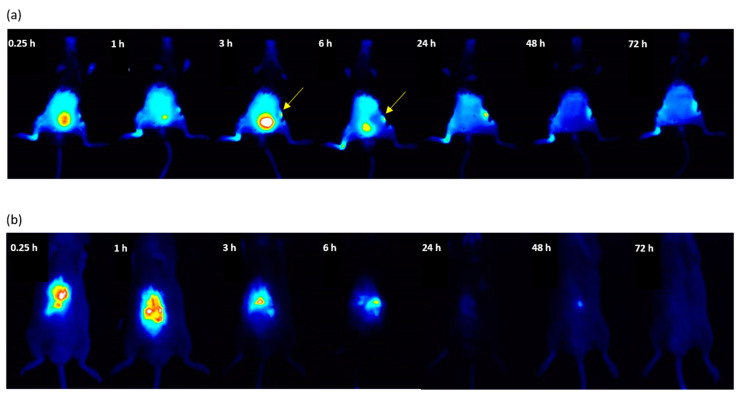
In vivo biodistribution of copolymer conjugate liquid chromatography (LC)-Cy5.5-DEX-OPB_i.v. (**a**) and fluorescent dye Cy 5.5, (**b**). Injection of the 0.1 mg Cy5.5 eq/kg, *n* = 6. The yellow arrows signify the copolymer conjugate accumulation in the inflamed skin after irritation by depilation and scratching.

A similar trend in biodistribution was observed after i.v. injection of polymer-drug conjugate LC-Cy5.5-DEX-OPB (Figure 3a). Dexamethasone conjugation to the polymer carrier did not influence its elimination by kidneys since the signal from the bladder for LC-Cy5.5-DEX-OPB also disappeared after 24 h. The time course of the polymer conjugate accumulation in arthritic joints (right) exhibits a trend comparable to the polymer carrier itself. The ability of the polymer to accumulate in inflamed tissues was also confirmed by the fluorescent signal from irritated skin in the abdomen, where the mice were depilated and scratched themselves (Figure 3a, see yellow arrow).

In the control group treated with free Cy5.5, the fluorescent dye did not accumulate in the affected tissue (right paw). Compared to polymer systems, the free dye accumulated in the liver within 15 min after administration, with most Cy5.5 eliminated from the liver within 24 h (Figure 3b).

**Figure 4:**
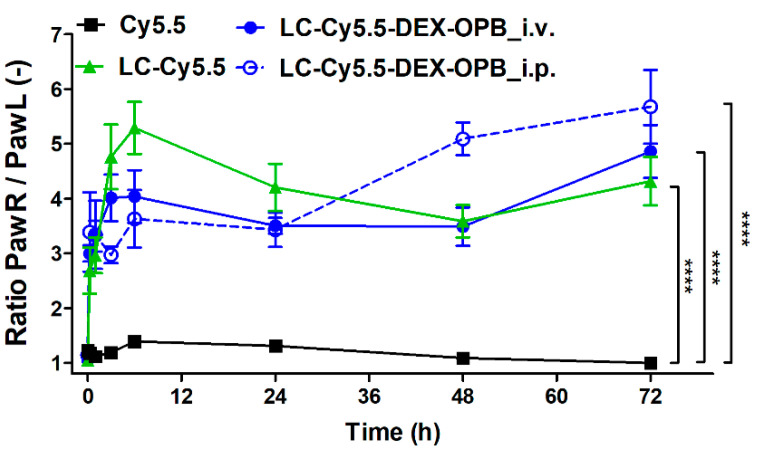
In vivo fluorescence data. The ratio of the arthritic right paw fluorescence to the healthy left paw fluorescence of the free fluorescent dye (Cy5.5), the fluorescently labeled linear copolymer (LC-Cy5.5) and fluorescently labeled linear copolymer conjugated to the ester of dexamethasone injected either i.v. (LC-Cy5.5-DEX-OPB_i.v.) or i.p. (LC-Cy5.5-DEX-OPB_i.p.). Injection of the 0.1 mg Cy5.5 eq/kg, *n* = 6.

Average normalized fluorescence in arthritic paws in Cy5.5, LC-Cy5.5, and LC-Cy5.5-DEX-OPB i.v. groups are displayed in Figure 4, providing a ratio of the accumulation rate in the inflamed joint compared to the control joint. At time point 0 (before injection), this ratio was equal to 1 in all experimental groups. It is of note that in the case of the Cy5.5 group, this ratio is also equal to 1 for the whole experiment, highlighting the absence of specific uptake in the arthritic paws of free Cy5.5 as a model of low molecular weight drug. Moreover, the low signal in both paws compared to highly fluorescent liver suggests little or no dye accumulation in joints. In contrast, when polymers LC-Cy5.5 or LC-Cy5.5-DEX-OPB_i.v. or LC-Cy5.5-DEX-OPB_i.p. were administered, we observed a considerably higher fluorescence signal from the arthritic paw as opposed to the healthy paw. For the LC-Cy5.5 conjugate, the maximum signal in the right paw was observed after 6 h, when the amount of the polymer in arthritic paw was almost 6 times higher in comparison to the healthy paw. Subsequently, the amount of signal decreases as the polymer is eliminated from the paw, possibly through lymphatic drainage.

Similar to LC-Cy5.5, the LC-Cy5.5-DEX-OPB conjugate administered i.v. accumulated rapidly in the arthritic paw, with a signal detected 15 min after application. Afterwards, the accumulation ratio slightly increased until the end of the experiment and was slightly different from the corresponding drug-free polymer-carriers with no sharp peak at 6 h. Due to the presence of the hydrophobic drug, the systemic circulation of LC-Cy5.5-DEX-OPB after i.v. administration might be altered and prolonged, e.g., by its interaction with plasmatic proteins or slower renal elimination [37,38] (see the results from ex vivo biodistribution in Section 3.2.2). However, the difference in accumulation rate between the two polymer systems was considered nonsignificant. In the last two time points of the experiment (48 and 72 h), the ratio was almost the same for both experimental groups.

The LC-Cy5.5-DEX-OPB was administered intravenously or intraperitoneally since the route of administration could influence its biodistribution and consequently the final therapy outcome. [39,40,41,42] When repeated doses are required, the daily i.v. administration to mice is limited due to the difficulty to repeat daily tail vein injection, as the tail vein is damaged after each injection, so it becomes difficult to ensure that the correct amount of substance has been delivered. In contrast, i.p. injection is easily applicable and less stressful for the mice. Moreover, in the case of higher polymer dosing, the i.v. injection becomes impossible due to the high viscosity of the applied solution. Both routes of administration of LC-Cy5.5-DEX-OPB followed a similar biodistribution trend (accumulation in inflamed paw, excretion by kidneys) and the ratio PawR/PawL was not significantly different between both administration routes up to the 24 h after the injection. Surprisingly, within 48 h and 72 h, the accumulation rate in the inflamed joint was substantially higher by i.p. than after i.v. administration (Figure 4), suggesting that clinically applicable routes alternative to i.v. such as subcutaneous injection would be a suitable option in humans.

The content of circulating LC-Cy5.5-DEX-OPB, the fluorescence signal from the vein of the left paw without the inflammation, was compared after i.v. or i.p. administration, see Figure S3. As expected, the major difference in the polymer content between i.v. or i.p. the administration was observed at the early time points, i.e., 15 min (*p* ≤ 0.001) and 1 h (*p* ≤ 0.01). After a direct injection to the bloodstream, the fluorescence signal in the vein of the healthy paw occurred immediately and decreased until the end of the experiment.

In summary, both polymers, LC-Cy5.5, and LC-Cy5.5-DEX-OPB were eliminated from the organism via the renal route, accumulating in arthritic joints to a considerably higher extent than the free dye (*p* ≤ 0.0001). No accumulation of the fluorescent dye used as a control was observed in the arthritic paw.

No significant weight loss in mice injected with LC-Cy5.5, LC-Cy5.5-DEX-OPB_i.p. or LC-Cy5.5-DEX-OPB_i.v. could be observed compared to the control group, providing good evidence of the absence of acute toxicity of these compounds. A decrease in weight of around 10% was observed in every experiment, which is characteristic of this arthritis model due to the highly painful state and stress (Figure S4).

**Figure 5:**
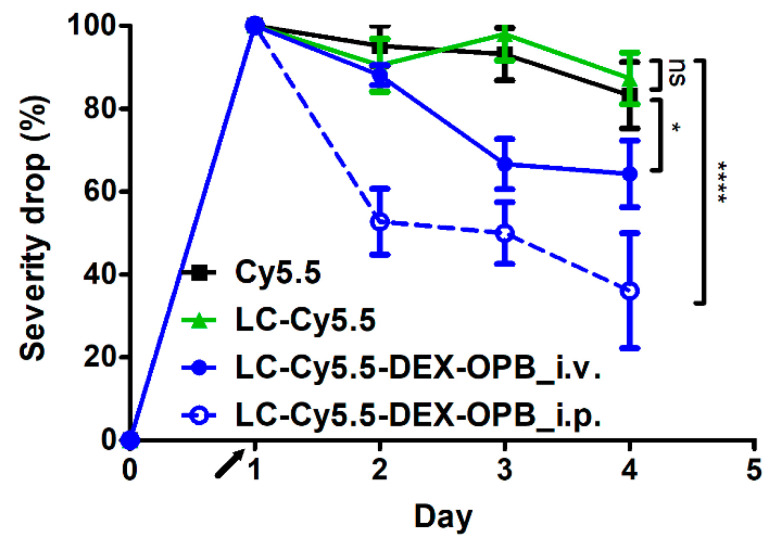
Decrease in the clinical severity (CS) of the inflammation in the mouse ankle in the experiment of biodistribution. Injection of the 0.1 mg Cy5.5 eq/kg and 0.6 mg DEX-OPB/kg, *n* = 6.

The biodistribution study was supported by CS observation of arthritic mice, which was correlated to polymer biodistribution with the inflammation rate. A slight decrease in edema of the tarsus and ankle was observed in all experimental groups, which is a typical index of remission of adjuvant-induced arthritis [43]. The administration of polymer conjugate LC-Cy5.5-DEX-OPB_i.v. led to a significant decrease in inflammation (by 36%) in the ankle, where the principal inflammation outbreak occurs, while the administration of LC-Cy5.5-DEX-OPB_i.p. decreased the inflammation by 64% (Figure 5). The decrease in inflammation severity in the tarsus followed a similar trend as in the ankle (Figure S5). The largest decrease of the CS occurred after injection of LC-Cy5.5-DEX-OPB_i.p., even though the administered drug dose was five times lower (0.6 mg of DEX-OPB/kg equal to 0.43 mg of DEX eq./kg) compared to the free DEX dose (3 mg DEX/kg) used as a control in the therapeutic experiments described in the Section 3.2.3. This result proved the beneficial therapeutic efficacy of the LC-Cy5.5-DEX-OPB applied via i.p. administration, which enables prolonged circulation of the nanomedicine in the body and subsequent accumulation in the arthritic joint.

#### 3.2.2. Ex Vivo Biodistribution

**Figure 6:**
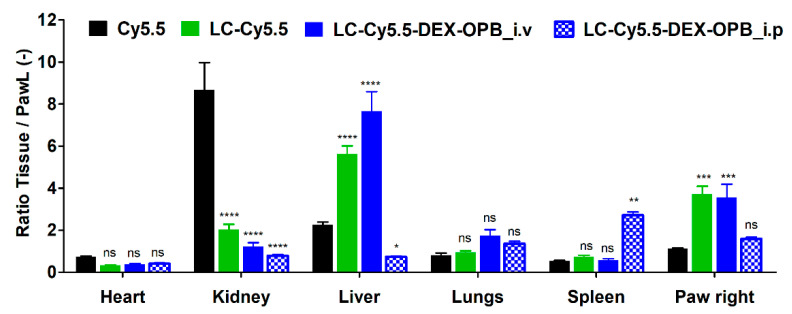
Ex vivo biodistribution of Cy5.5, LC-Cy5.5, and LC-Cy5.5-DEX-OPB injected i.v. or i.p. The ratio tissue/left paw is normalized to the weight of the given tissue. Injection of the 0.1 mg Cy5.5 eq/kg, *n* = 6.

The fluorescence intensity from each organ collected at 75 h post-injection was normalized for the weight of each organ and laser beam intensity. The elimination of free Cy5.5 and LC-Cy5.5 was faster in almost all organs in comparison to that observed with LC-Cy5.5-DEX-OPB_i.v. (Figure 6).

The highest content of free Cy5.5 was detected in the kidneys implying renal retention of free dye before elimination from the body. Almost no signal was found in the liver for free Cy5.5 in good agreement with in vivo fluorescence signal which disappeared after 72 h. The ratio between the right and left paw was equal to 1, confirming that there is no difference between the signal of the arthritic and the healthy paws.

In the case of LC-Cy5.5, the ratio of the signal between right and left paw was almost equal to 3, proving beneficial accumulation in the arthritic (right) paw. Ex vivo results suggest that the LC-Cy5.5 elimination from the body was faster since there was a lower signal in all organs when compared to that observed for LC-Cy5.5-DEX-OPB_i.v.

In almost all organs, the highest fluorescence signal was observed for LC-Cy5.5-DEX-OPB_i.v., thus showing that the polymer-drug conjugate was eliminated more slowly than its polymer precursor. Therefore, the conjugation of DEX-OPB appears to change the biodistribution of the polymer-drug carrier by prolonging its circulation. The higher fluorescent signal in the lungs was probably due to higher hydrophobicity of the drug-polymer conjugate compared to the drug-free polymer carrier. It is well-known that due to larger lung capillary surfaces, hydrophobic substances are retained in the lungs [44]. Similarly, the higher liver content of LC-Cy5.5-DEX-OPB_i.v. could be attributed to higher hydrophobicity of the conjugate with DEX-OPB. We hypothesize that the higher hydrophobic nature of the conjugate leads to enhanced interaction with hepatocytes, thus increased accumulation in the liver. The ratio of the signal between the arthritic and the left paw was equal to that of the experiment with LC-Cy5.5, suggesting that the accumulation rate in acute arthritis is predominantly influenced by the macromolecular size, less by its hydrophilic/hydrophobic balance.

The i.v. and i.p. administration of LC-Cy5.5-DEX-OPB led to the diverse biodistribution ex vivo, with differences mainly in the liver, spleen, and arthritic right paw (see Figure 6), especially concerning the nanomedicine concentration in the systemic blood circulation. Previous studies have demonstrated that i.v. injection of nanoparticles is followed by their rapid opsonization in plasma [42]. This biological phenomenon is accompanied by immediate uptake by spleen and liver macrophages [45]. Opsonization is mainly caused by plasma proteins, e.g., fibronectin, C-reactive protein, or type I collagen, whose blood concentrations are substantially increased during inflammation making nanomedicine accumulation more likely in the spleen and liver [46]. Due to this reason, we observed higher accumulation in the liver after direct i.v. administration. In the case of i.p. administration, the absorption rate depends on the physicochemical characteristics of the applied molecules [47]. The transfer from the peritoneal cavity into the bloodstream can occur via two possible routes, i.e., the substances are carried directly to the liver by portal or extra portal pathways of the mesenteric blood system [48]. The larger macromolecules are usually returned to the circulation by the lymphatic system [19] and we believe that this phenomenon is the cause of the observed increase of the LC-Cy5.5-DEX-OPB_i.p. accumulation in the spleen. Indeed, the higher therapeutic activity of LC-Cy5.5-DEX-OPB_i.p. leads to profound removal of immune cells from the site of inflammation, thus leading to the transfer of the polymer in these cells to the spleen.

The lower ratio of PawR/PawL for LC-Cy5.5-DEX-OPB_i.p. than for i.v. application is the result of the effectivity of the treatment, which leads to a decrease in the inflammation, i.e., lower inflammation score of the arthritic paw (64% decrease of CS for LC-Cy5.5-DEX-OPB_i.p. in comparison to 36% decrease of CS for LC-Cy5.5-DEX-OPB_i.v.). The decreased inflammation leads to a reduction in the size of the site of inflammation, thus to the lower amount of the polymer detected in the inflammation site.

#### 3.2.3. In Vivo—Anti-Inflammatory Activity

The therapeutic effect of the linear conjugate LC-Cy5.5-DEX-OPB was studied to prove its anti-inflammatory ability and to compare its efficacy to the standard treatment of acute exacerbation of arthritis and other CID by parenteral administration of free DEX [49]. The CS of the right paw (ankle and tarsus) was studied using two different dosing regimens: i) 3 mg DEX or DEX-OPB/kg once on day 1; ii) three times 1 mg DEX or DEX-OPB/kg on days 1, 2, and 3. Moreover, the two routes of administration, i.p and i.v., of polymer conjugate were compared. The health status of mice was observed daily with the emphasis on mouse weight, locomotion, and activity in the cage, ruffled fur, and any additional pathologies. The mice treated with the LC-Cy5.5-DEX-OPB or DEX were more active with a good condition of the fur than the non-treated control. Indeed, the weight decrease associated with the pain and stress of the mice was observed in all test groups as in the case of the biodistribution experiment. Thus, the weight fluctuation in this model is not only associated with the toxicity of applied systems but also with their therapeutic activity. With growing inflammation in hind paws, the food intake in affected mice decreases.

**Figure 7:**
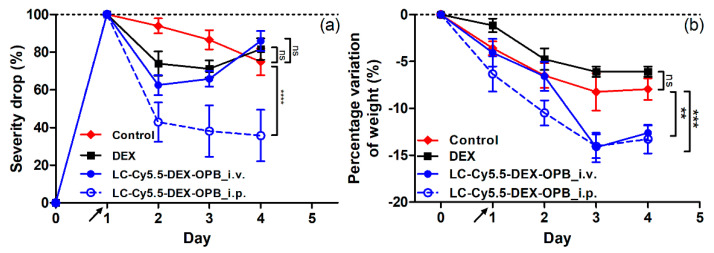
Decrease of the CS of the inflammation of mice in the ankle (**a**) and decrease of the weight (**b**) of the mice in the therapeutic experiment with dosing 1 × 3 mg DEX-OPB/kg. *n* = 6.

The application of LC-Cy5.5-DEX-OPB in the single i.v. or i.p. dosing of 3 mg DEX or DEX-OPB/kg led to a sharp decrease of the edema of the ankle within 24 h–LC-Cy5.5-DEX-OPB_i.v. (CS decrease by 38% in ankle) and LC-Cy5.5-DEX-OPB_i.p. (CS decrease by 64% in ankle), compared to less pronounced decrease (CS decrease by 29% in ankle) induced by free DEX (Figure 7a). The LC Cy5.5-DEX-OPB_i.v. treatment did not further reduce the inflammation status, and the ankle edema slightly increased within 48 and 72 h. This was associated with the elimination of most DEX-bearing polymer from the organism within 24 h after administration. A similar trend was observed in the case of therapy with free DEX, where the CS stagnates within 48 h and 72 h. The most dramatic CS decrease was observed with LC-Cy5.5-DEX-OPB_i.p. treatment and its therapeutic effect remained until the end of the experiment. The injection into the peritoneal cavity improved the therapy outcome of the polymer conjugate due to the prolonged circulation of the nanomedicine in the organism, with both i.v. and i.p. administration of LC-Cy5.5-DEX-OPB in higher single dose causing a small weight drop (up to 15% of weight decrease) in comparison to the control group (up to 8% of weight decrease) showing partial toxicity in mice (see Figure 7b). Therefore, we decided to divide the single dose into multiple doses administered sequentially over three days.

**Figure 8:**
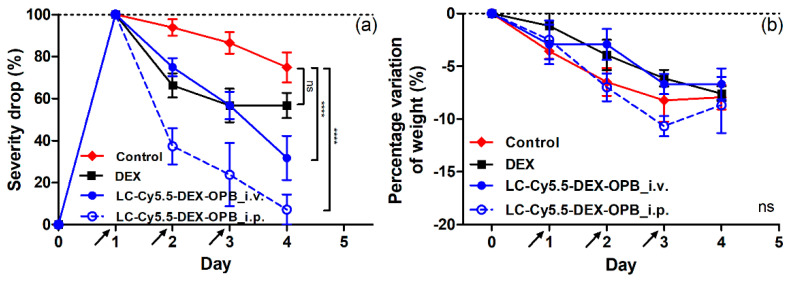
Decrease of the CS of the inflammation of mice in the ankle (**a**) and decrease of the weight (**b**) of the mice in the therapeutic experiment with dosing 3 × 1 mg DEX or DEX-OPB/kg. *n* = 6.

The second dosing regimen (3 × 1 mg DEX or DEX-OPB/kg i.v. or i.p. daily) was more successful with a maximal decrease of the CS of the inflammation (CS decrease by 93% in ankle) observed for LC-Cy5.5-DEX-OPB_i.p. treatment (Figure 8a). Analogously to the single-dose regimen, the i.v. application of LC-Cy5.5-DEX-OPB (CS decrease by 68% in ankle) was less effective than the equal i.p. dosing, however, substantially more effective than the treatment with DEX alone (CS decrease by 43% in ankle). All treated groups showed a continuous decreasing trend of inflammation status throughout the experiment (72 h), unlike the single-dose regimen. CS decrease was observed in both parts of the arthritic leg and, for both applications of polymer conjugate, the therapeutic effect was significantly higher than that for free DEX. In the tarsus, where the inflammation is less pronounced than in the ankle or not developed in all cases, the trend of inflammation decrease was comparable to the ankle, unsurprisingly, with repeated applications of LC-Cy5.5-DEX-OPB_i.p. being the most efficient treatment. (Figure S6). Here, in the multiple-dose regimen, both i.v. and i.p. administration of LC-Cy5.5-DEX-OPB did not cause any significant difference in the weight drop in comparison to control mice, suggesting that it had no acute toxic effects (Figure 8b). The application of free DEX in single or multiple regimens led to a comparable weight decrease.

The anti-inflammatory activity was also determined by the amount of ROS in inflamed tissue (right paw) using bioluminescence of 3-aminophthalhydrazide monosodium salt, see Supplementary Materials and Figure S7. No significant ROS overproduction was observed in the one-day or three-day application scheme of LC-Cy5.5-DEX-OPB (injected i.v. or i.p.), thus proving that no such side effects are presented during the polymer-DEX-OPB treatment.

In summary, polymer-DEX conjugate had beneficial properties, especially in the three-day dosing scheme, showing both significant therapeutic efficacy and no toxicity-connected reduction of weight loss.

## 4. Conclusions

Here, we describe the synthesis and study of the physicochemical and biological properties of linear pHPMA conjugates with the fluorescent dye and the anti-inflammatory drug dexamethasone bound via a stimuli-responsive spacer. The pHPMA were prepared by RAFT polymerization yielding well-defined copolymers with the required molar mass and very low dispersity (*Đ* = 1.05). These copolymers were used for the synthesis of polymer conjugates, labeled with the fluorescent dye Cy5.5 attached via a physiological stable hydrazide bond. The dexamethasone derivative DEX-OPB was attached via a pH-sensitive hydrazone bond enabling rapid release from the polymer at the pH modeling the environment inside immune cells. DEX-OPB was rapidly transformed to DEX in the presence of carboxyesterase, to which the polymer conjugate is exposed after uptake by immune cells. The fluorescently labeled conjugates with/without drug accumulated in the inflamed leg compared to the healthy control using a murine model of adjuvant-induced acute arthritis. The accumulation occurred rapidly after polymer injection and was maintained until the end of the experiment. The intraperitoneal administration of LC-Cy5.5-DEX-OPB led to a superior therapeutic outcome in all dosing regimens tested, with the maximal therapeutic effect observed by i.p. injection of a dose of 1 mg DEX or DEX-OPB/kg on day 1, day 2, and day 3. Surprisingly, the therapeutic effect of the LC-Cy5.5-DEX-OPB injected i.p. was also observed after the injection of a very low single dose of 0.6 mg/kg DEX-OPB. Since the polymer conjugates have proved to accumulate in the inflamed tissues and exhibit superior anti-inflammatory effect compared to low molecular weight drugs, we strongly believe that our polymer conjugates have a great potential in the treatment of rheumatoid arthritis and other joint inflammation-related diseases.

## Figures and Tables

**Table 1:** Characterization of copolymer precursors.

Copolymer	*M*_w_^a^ (kg/mol)	*Đ*^a^ (-)	Mon:CTA:Ini Ratio	Hydrazides (mol %)	*R*_H_^c^ (nm)	*F*_TTc_^d^ (-)
LC-TTc	33	1.05	500:2:1	-	4.0	0.98
LC	33	1.05	-	-	4.0	-
LC-NHNH_2_	37	1.11	-	6.0	4.5	-

^a^ Molecular weight and dispersity were determined by SEC using the TSKgel SuperAW3000 column and RI and MALS detection; ^b^ The content of deprotected hydrazides was determined by TNBSA; ^c^ The hydrodynamic radius was determined by DLS; ^d^ Polymer functionality of the TTc end groups (the ratio between the Mn value determined by SEC and the Mn value calculated from the content of the TTc end-chain groups determined by spectrophotometry).

**Table 2:** Characteristics of polymer conjugates.

Sample	*M*_w_^a^ (kg/mol)	*Đ*^a^ (-)	Cy 5.5 ^b^ (*wt* %)	DEX-OPB ^c^ (*wt* %)	*R*_H_^d^ (nm)	*PDI* ^d^
LC-DEX-OPB	39	1.1	-	5.2	5.0	0.3
LC-Cy5.5	37 ^*^	1.1 ^*^	1.75	-	4.5 ^*^	0.3 *
LC-Cy5.5-DEX-OPB	37 ^*^	1.1 ^*^	1.00	6.0	4.5 ^*^	0.3 *

^a^ Molecular weight and dispersity were determined by SEC using the TSKgel SuperAW3000 column and RI and MALS detection; ^b^ The content of Cy5.5 was determined spectrophotometrically; ^c^ The content of DEX-OPB was determined by HPLC; ^d^ The hydrodynamic radius was determined by DLS. * Characteristics of the polymer precursor without Cy5.5 and DEX-OPB.

## Supplementary Materials

The following are available online at https://www.mdpi.com/1999-4923/12/8/700/s1, Figure S1: Reaction scheme of the preparation of the polymer conjugate with the fluorescent dye (Cy5.5) and derivate of dexamethasone (DEX-OPB). Figure S2: Disappearance of the UV/VIS signal of the TTc functional group of the polymer precursor LC-TTc (black) and the polymer precursor LC after reaction with AIBN (red). Figure S3: In vivo fluorescence data from the healthy leg of the linear copolymer with the fluorescent dye and the dexamethasone injected i.v (LC-Cy5.5-DEX-OPB_i.v.) or i.p (LC-Cy5.5-DEX-OPB_i.p.). Injection of the 0.1 mg Cy5.5 eq/kg, *n* = 6. Figure S4: Decrease in the weight of the mice during the biodistribution experiment. Injection of the 0. 1 mg Cy5.5 eq/kg, *n* = 6. Figure S5: Decrease of the clinical severity (CS) of the inflammation of the mice, tarsus, during the biodistribution experiment. Injection of the 0.1 mg Cy5.5 eq/kg, *n* = 6. Figure S6: Decrease of the CS of the inflammation of mice, tarsus, in the therapeutic experiment of the DEX and the LC-Cy.5.5-DEX-OPB injected i.v. and i.p. in the experiment with two different dosings 1 × 3 mg DEX eq/kg (a) and 3 × 1 mg DEX eq/kg. (b). *n* = 6. Figure S7: Activity of ROS in two experiments with different dosing. (a) 1 × 3 mg DEX eq/kg; (b) 3 × 1 mg DEX eq/kg. *n* = 6.
