# Supplementary material for: Polymer Nanomedicines with Ph-Sensitive Release of Dexamethasone for the Localized Treatment of Inflammation
Source: Pharmaceutics. 2020 Jul 25;12(8):700. doi: 10.3390/pharmaceutics12080700 (PMC7465548; doi:10.3390/pharmaceutics12080700)
Supplement: Supplementary file 1 [file pharmaceutics-12-00700-s001.pdf]

pharmaceutics

Supplementary Materials: Polymer Nanomedicines with Ph-Sensitive Release of Dexamethasone for the Localized Treatment of Inflammation

Alena Libánská, Eva Randárová, Franck Lager, Gilles Renault, Daniel Scherman and Tomáš Etrych *

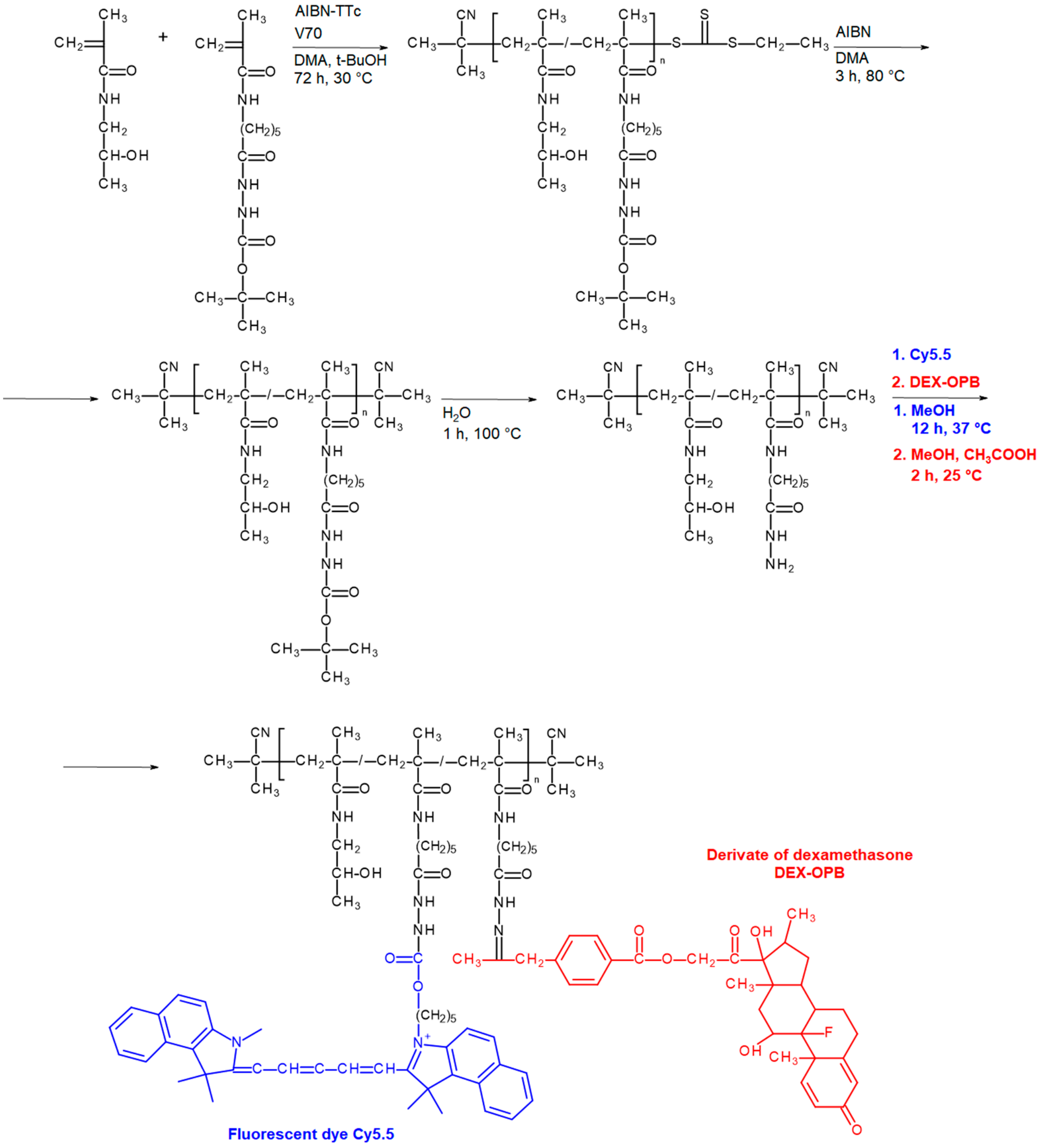

Figure S1. Reaction scheme of the preparation of the polymer conjugate with the fluorescent dye (Cy5.5) and derivate of dexamethasone (DEX-OPB).

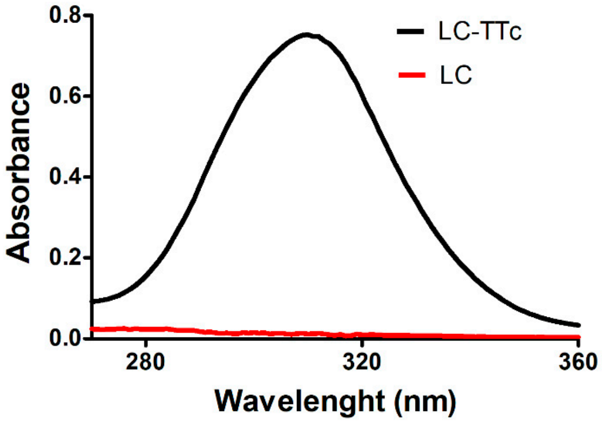

Figure S2. Disappearance of the UV/VIS signal of the TTc functional group of the polymer precursor LC-TTc (black) and the polymer precursor LC after reaction with AIBN (red).

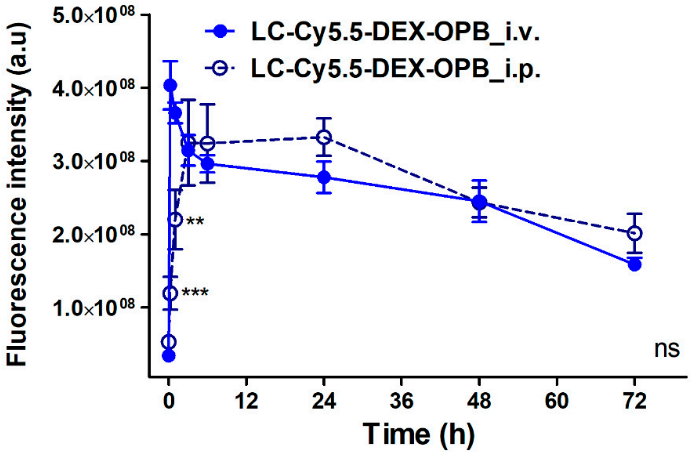

Figure S3. In vivo fluorescence data from the healthy leg of the linear copolymer with the fluorescent dye and the dexamethasone injected i.v (LC-Cy5.5-DEX-OPB_i.v.) or i.p (LC-Cy5.5-DEX-OPB_i.p.). Injection of the 0.1 mg Cy5.5 eq/kg, n = 6.

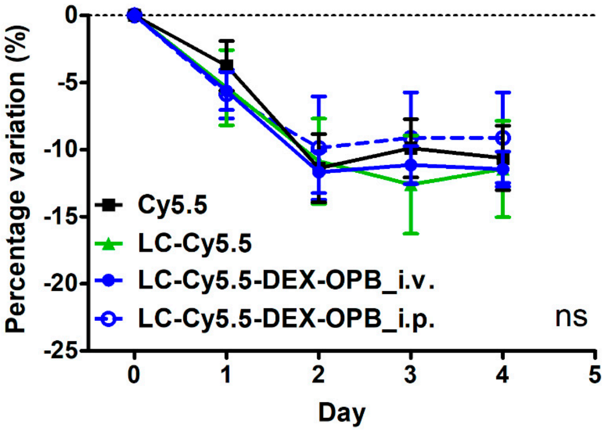

Figure S4. Decrease in the weight of the mice during the biodistribution experiment. Injection of the 0. 1 mg Cy5.5 eq/kg, n = 6.

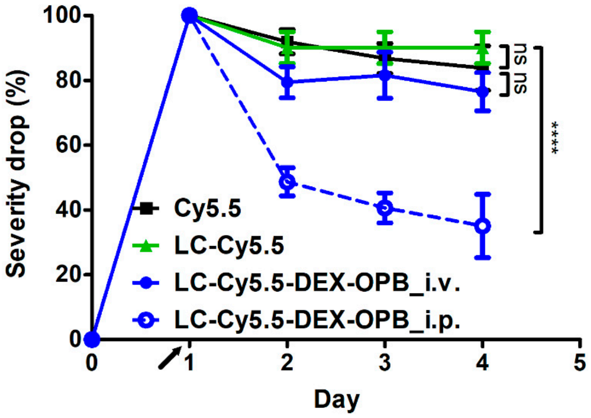

Figure S5. Decrease of the clinical severity (CS) of the inflammation of the mice, tarsus, during the biodistribution experiment. Injection of the 0.1 mg Cy5.5 eq/kg, *n* = 6.

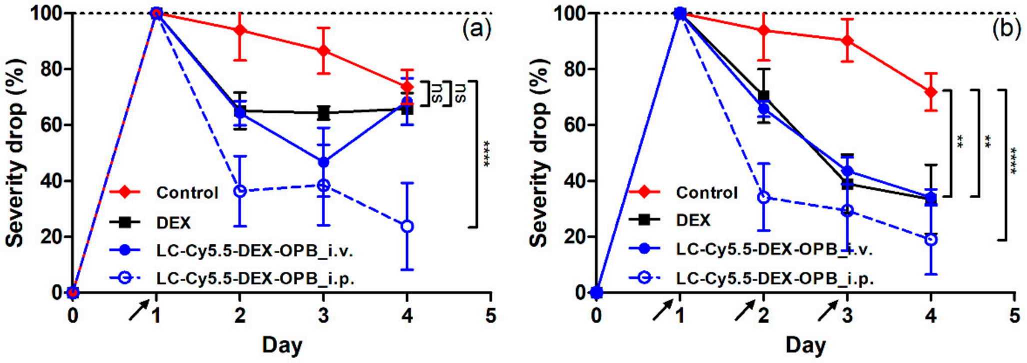

Figure S6. Decrease of the CS of the inflammation of mice, tarsus, in the therapeutic experiment of the DEX and the LC-Cy.5.5-DEX-OPB injected i.v. and i.p. in the experiment with two different dosings 1 × 3 mg DEX eq/kg (**a**) and 3 × 1 mg DEX eq/kg. (**b**) *n* = 6.

ROS determination

One key feature of the synovial joints in RA is a higher ROS content detected in affected tissues compared to normal tissue [1]. The excessive amount of ROS promotes the production of cytokines, such as TNF-α, IL-1β, and IL-6, and the interactions between inflammatory cytokines and ROS would accelerate RA progression [2]. Moreover, elevated levels of ROS in RA can activate metalloproteinases, inhibit cartilage proteoglycan synthesis, promote chondrocyte apoptosis, ultimately leading to disruption of the cartilage and bone tissue [2].

The measurement of the bioluminescence originating from the reaction of the luminol with ROS of neutrophils was performed daily to monitor the therapy course, see Figure SI7. In the one-day dosing scheme, no significant differences between treated groups were observed. However, the daily injection of the DEX showed a significant increase in the signal with a peak after 48 h. This effect could be caused by the increased output of the neutrophils [3], a side effect of the treatment with free DEX. The application of LC-Cy5.5-DEX-OPB (injected i.v. or i.p.) does not exhibit such side effects. The detailed effect of polymer conjugate on healthy tissues will be studied further.

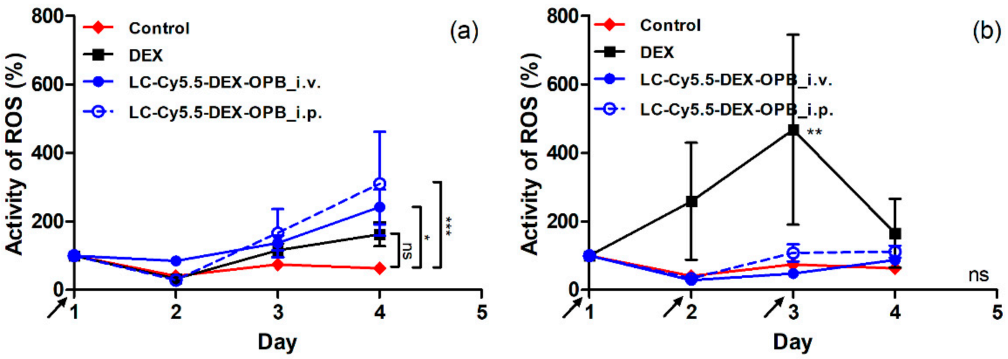

Figure S7. Activity of ROS in two experiments with different dosing. (**a**) 1 × 3 mg DEX eq./kg; (**b**) 3 × 1 mg DEX eq/kg. $n = 6$.

References

1. Davis 4th, R.W.; Snyder, E.; Miller, J.; Carter, S.; Houser, C.; Klampatsa, A.; Albelda, S.M.; Cengel, K.A.; Busch, T.M. Luminol Chemiluminescence Reports Photodynamic Therapy-Generated Neutrophil Activity In Vivo and Serves as a Biomarker of Therapeutic Efficacy. *Photochem. Photobiol.* **2019**, *95*, 430–438, doi:10.1111/php.13040.
2. Ni, R.; Song, G.; Fu, X.; Song, R.; Li, L.; Pu, W.; Gao, J.; Hu, J.; Liu, Q.; He, F.; et al. Reactive oxygen species-responsive dexamethasone-loaded nanoparticles for targeted treatment of rheumatoid arthritis via suppressing the iRhom2/TNF-α/BAFF signaling pathway. *Biomaterials* **2020**, *232*, 119730, doi:10.1016/j.biomaterials.2019.119730.
3. Ohkaru, Y.; Arai, N.; Ohno, H.; Sato, S.; Sakakibara, Y.; Suzuki, H.; Aritoshi, S.; Akimoto, S.; Ban, T.; Tanihata, J.; et al. Acute and subacute effects of dexamethasone on the number of white blood cells in rats. *J. Heal. Sci*. **2010**, *56*, 215–220, doi:10.1248/jhs.56.215.
